# Response of Copepods to Elevated *p*CO_2_ and Environmental Copper as Co-Stressors – A Multigenerational Study

**DOI:** 10.1371/journal.pone.0071257

**Published:** 2013-08-07

**Authors:** Susan C. Fitzer, Gary S. Caldwell, Anthony S. Clare, Robert C. Upstill-Goddard, Matthew G. Bentley

**Affiliations:** 1 School of Marine Science and Technology, Newcastle University, Newcastle upon Tyne, United Kingdom; 2 School of Geographical and Earth Sciences, Glasgow University, Glasgow, United Kingdom; Dowling College, United States of America

## Abstract

We examined the impacts of ocean acidification and copper as co-stressors on the reproduction and population level responses of the benthic copepod *Tisbe battagliai* across two generations. Naupliar production, growth, and cuticle elemental composition were determined for four pH values: 8.06 (control); 7.95; 7.82; 7.67, with copper addition to concentrations equivalent to those in benthic pore waters. An additive synergistic effect was observed; the decline in naupliar production was greater with added copper at decreasing pH than for decreasing pH alone. Naupliar production modelled for the two generations revealed a negative synergistic impact between ocean acidification and environmentally relevant copper concentrations. Conversely, copper addition enhanced copepod growth, with larger copepods produced at each pH compared to the impact of pH alone. Copepod digests revealed significantly reduced cuticle concentrations of sulphur, phosphorus and calcium under decreasing pH; further, copper uptake increased to toxic levels that lead to reduced naupliar production. These data suggest that ocean acidification will enhance copper bioavailability, resulting in larger, but less fecund individuals that may have an overall detrimental outcome for copepod populations.

## Introduction

The geological record reveals several periods of past acidification of the global ocean [1] that coincide with marine mass extinctions [2]–[3]. The inferred decline in ocean pH since the industrial revolution is far more rapid than previously observed [4] and is predicted to continue under ‘business as usual’ CO_2_ emission scenarios [5]. Indeed, the current acidification may be one of the most significant environmental features of the Anthropocene [6]–[9], with the most extreme predictions giving a 0.4 pH unit decline by 2100 [4], [10]. The potential impacts on ecosystem health and function, particularly when extrapolated to subsequent effects on ecosystem services, are severe [11]–[13]. Nevertheless, there remain substantial uncertainties regarding pH responses at the individual species level.

While the early life histories of several marine organisms have been found to be vulnerable to ocean acidification [14]–[15], comparatively few studies have examined the long-term, multi-generational impacts that are important for predicting possible future adaptations [16]–[18]. However, it is important to recognise that ocean acidification is only one aspect of global change [19] and that synergistic effects involving other variables in combination with pH must also be considered. One such example is the existence of elevated levels of xenobiotics. Metal contaminants are a particular concern for coastal ecosystems, especially soft sediment habitats where metals typically accumulate to greater concentrations than in the overlying water [20]–[21]. Differences in toxicity have been related to differences in metal binding sites between bottom waters, sediment pore water, suspended particles and sediments [22]–[23]; for example, cadmium and copper are most toxic in sediment pore waters [22]–[23]. Toxicity tests involving sediment-bound copper revealed enhanced copper mobilisation at pH 4 relative to pH 7 [21]. As pH decreases, copper bioavailability increases by the increase in free copper ion concentration [24], and hence toxic effects may be encountered. Indeed, Richards et al. 2011 [25] predicted an increase in free copper ions of 115% over the next 100 years in estuarine environments as a result of declining seawater pH and increasing temperature. Therefore, ocean acidification may potentially exacerbate the toxic effects of copper in meiobenthic communities and may thus prove problematic for the fitness of benthic organisms.

Copepods have been widely utilised in bioassays for metal toxicity, and increasingly also for ocean acidification studies; however, copepods have been shown to have a high tolerance for copper as one of the essential trace metals [26]. Previous studies combining the effects of ocean acidification with a further environmental stressor have lacked the predictive power that a multi-generational approach affords [27]–[37]. We utilised the harpacticoid copepod *Tisbe battagliai* as a test species. *Tisbe* is well suited to multigenerational studies due to its ease of culture, rapid life cycle and pedigree in ecotoxicology studies; including assessing the impacts of ocean acidification [16], [21], [38]–[42]. Harpacticoid copepods are an integral component of meiofaunal communities. They have a primarily benthic lifestyle, feeding on microalgae and detritus near, or at the sediment-water interface but can also be tychopelagic. The close association with marine sediments means that harpacticoids are subjected to lifelong exposure to elevated trace metals [22]. In this study we investigated the combined effects of ocean acidification and copper on the reproductive output, somatic growth and cuticle composition of *T. battagliai*.

## Materials and Methods

### Animal Husbandry and Experimental Exposure

Stock copepod cultures were maintained at 19°C, pH 8.06 and a 12∶12 L:D photoperiod and fed *ad libitum* on a mixed microalgae diet of *Isochrysis galbana* (4.4×10^5^ cells ml^−1^) and *Tetraselmis suecica* (2.0×10^5^ cells ml^−1^) [16]. The carbonate system parameters are given in Table 1, including the error on the pH, and were calculated as per Fitzer et al. 2012 [16]. The system used injected a minimal, yet unknown amount of CO_2_ directly into a constant air flow, which was monitored and controlled by glass pH probes via a solenoid valve and an automated Aqua-medic™ pH computer. Although it is noted that there can be potential problems with progressive divergence in the liquid junction potential between buffer and sample at estuarine salinities using glass electrodes to monitor pH [43], the relatively small salinity range observed during our experiments would suggest that this is of minor concern. Measurements of temperature and salinity were taken daily using standard protocols, which were then added to the logged pH values (Dr DAQ™ data logger) and total alkalinity (TA) data (closed titrations) to calculate the carbonate parameters as per the ocean acidification guidelines using CO2Sys [44]. Single, gravid females were placed in individual wells in modified 12-well plates [16]. Experimental pH values were 7.67±0.02, 7.82±0.02, 7.95±0.02 and 8.06±0.06 (control) (errors presented ± one standard deviation).

**Table 1:** pH and carbonate parameters maintained throughout experiments.

pH	Generation	Mean pH	Meantemperature(°C)	Salinity		Mean TA(CaCO_3_ mg L^−1^)	HCO_3_ in(µmol kg^−1^ SW)	CO_3_ in(µmolkg^−1^ SW)	*P*CO_2_ in(µatm)	ΩCa out	ΩAr out
8.06	G0	8.02±0.02	18.63±0.33		36.4	1347	1061	100	236	2.38	1.54
8.06	G1	8.10±0.06	18.53±0.34		37.6	1356	1017	118	188	2.78	1.80
7.95	G0	7.95±0.02	18.93±0.33		37.0	1279	1033	85	270	2.00	1.30
7.95	G1	7.93±0.03	18.62±0.18		38.0	1339	1092	86	298	2.02	1.31
7.82	G0	7.81±0.02	18.80±0.52		37.0	1312	1122	66	405	1.57	1.02
7.82	G1	7.81±0.03	18.86±0.52		37.0	1292	1103	66	398	1.55	1.01
7.67	G0	7.66±0.03	18.43±0.36		37.7	1359	1213	51	616	1.20	0.78
7.67	G1	7.66±0.03	18.43±0.36		36.0	1252	1121	46	571	1.08	0.70

pH, temperature, salinity and total alkalinity (TA) were all measured to calculate hydrogen carbonate (HCO_3_^−^), carbonate (CO_3_^2−^), partial pressure of carbon dioxide (*P*CO_2_), calcite (ΩCa) and aragonite (ΩAr) using CO_2_Sys software. Errors presented are one standard deviation from the mean.

### Environmental Copper Simulation Using Environmentally Relevant Concentrations

Pore water and seawater concentrations of copper were determined on samples collected at low tide from three locations at Black Middens (GPS Latitude: 55° 1.557’N, Longitude: 1° 25.549’W) in the lower Tyne estuary, UK. Sediment samples (∼70 g) were centrifuged in 50 ml centrifuge tubes at 3000 rpm (996 RCF) for 1 min. Permits and approvals to collect samples were not required from North Tyneside Council as the samples were collected from public access beaches in Tyne and Wear, UK; these sites were also free from endangered or protected species. The separated pore waters were then removed using disposable 1 ml syringes and filtered through 0.45 µm cellulose nitrate membranes. Samples were subsequently split between two 30 ml acid washed glass bottles (10% nitric acid) and fixed with 1 ml of 0.05 M nitric acid (Analar). They were then stored at 4°C prior to analysis by inductively coupled plasma mass spectroscopy (ICP-MS) at a UKAS accredited laboratory (Environmental Scientifics Group Limited).

Average pore water copper concentrations were 6 µg L^−1^, which is broadly similar to previously published pore water values of 10–14 µg L^−1^ found in European *Tisbe* habitats [45]–[48]. These concentrations were subsequently used to benchmark copper spiked experimental seawater. 1 ml of a copper stock solution (330 mg CuSO_4_ L^−1^ in Milli-Q water) was added to 6 L of filtered natural seawater (salinity 36, collected Tyne estuary, UK); CuSO_4_ is often used in copper toxicity testing [39], [49]. Addition of the pore water spike to natural seawater (copper) at 14 µg L^−1^ gave a final concentration of 20 µg L^−1^.

### Measuring Naupliar Production and Growth in Tisbe Battagliai

Gravid females (N = 32) were pipetted into wells in three floating 12-well plates held within an experimental tank at the appropriate pH. Naupliar production was recorded daily. Nauplii were removed and fixed daily for growth determination and elemental analysis of the cuticle. Samples were fixed using a 2.5 : 1 mixture of 40% formaldehyde and 25% gluteraldehyde. Sodium phosphate (84 mM) and sodium hydroxide (67.5 mM) were added as buffers (McDowell and Trumps fixative). This fixation protocol was also suitable for subsequent electron microscopy [50]. Following the initial collection of naupliar production data for three broods per female, the progeny from the first brood were grown on to adults and the initial maternal females removed; these produced the subsequent generation of gravid females. To take account of brood, variability data were collected across three broods (∼ 12 days) [51]. This was repeated three times with the first generation discounted to allow for acclimation of the copepods to each pH. Subsequent generations were labelled G0 and G1.

For each naupliar stage in the first brood for each pH, growth was measured using an inverted dissection microscope (Olympus CHx41 at × 40 and × 4 objective) coupled with a digital camera (Sanyo) and ImageJ software.

Environmental scanning electron microscopy (ESEM) coupled with energy dispersive X-ray (EDX) analysis was used to determine cuticle elemental composition (% by weight) of fixed, air-dried samples (FEI XL30 ESEM-FEG coupled with Quantac EDX system manufactured by Rontec). Gravid females were used from the second generation for each pH. Three point analyses were performed on each of three copepods.

### Copepod Total Copper Concentration

Flame atomic absorption spectroscopy (FAAS) was used to determine the final concentration of copper within the copepod tissues. Owing to their small individual biomass and anticipated low copper concentrations [52] whole body digests were performed on batches of 10 individuals per replicate per treatment. Ten individual gravid females were sampled following the production of their third brood at the end of each generation of experiments at each pH. Copepods were fixed using McDowell and Trumps fixative. Three replicate batches were taken for each pH. Specimens were rinsed in deionised water, placed in pre-weighed glass tubes, dried at 25°C for 24 h and weighed to obtain dry weights. Specimens were digested in 100 µL of concentrated (70% AnalaR) nitric acid in a heat block at 60°C for 48 h. When complete the digests were diluted with deionised water to a volume of 3 ml prior to FAAS (Varian Spectra AA 50) analysis. The FAAS was calibrated using copper standards of 0, 2, 4, 6, 8 and 10 mg L^−1^ in nitric acid. Samples were injected into the FAAS at two second integrations.

### Statistical Analysis

Naupliar production data were analysed via the mixed modelling approach of generalised least squares (GLS). The model examined all interactions between the factors pH and generation, thereby eliminating pseudo-replication in the experimental design [53]–[55]. Correlation functions controlling pseudo-replication were calculated following the methodology of Gelman and Hill 2007 [56]. Data were modelled comparing each pH and generation of copepod to the intercept values of the control pH (8.06), and generation zero of copepods grown with addition of copper. Incorporating data from pH 8.06 to pH 7.67 through two generations of copepod within the model enabled a proxy for modelling data over the next 100 years of predicted ocean acidification. The model was assessed using the maximum likelihood (ML) of the data fit, incorporating Akaike’s information criterion (AIC) to assess the model of best fit. A second model was produced incorporating data for naupliar production without the addition of copper taken from our previous work [16]. Naupliar production data were modelled comparing all pHs with and without addition of copper from generation G0 to G1 to the intercept of the control pH (8.06) with addition of copper. Data for generation three (G2) of naupliar production without the addition of copper were omitted for the purposes of the new model [16]. Growth data were also analysed using the GLS mixed modelling approach. The data were modelled comparing all pHs and stages C4 - N3 to the intercepts of the control pH (8.06) and stage C5. Generation comparisons were included in the model with G0 (the earliest generation of all pH data) compared to all pHs and G1. This approach allowed for the analysis of the correlation factors within the experimental design so as to assess all interactions and eliminate any possible pseudo-replication. All modelling analysis was done using the ‘R’ platform R.Gui 2.9.1.

Cuticle elemental composition was analysed using analysis of variance (ANOVA) with all pHs being compared to the control pH 8.06 for each element of interest. Analysis was undertaken using Minitab15.

## Results

### Naupliar Production

**Figure 1:**
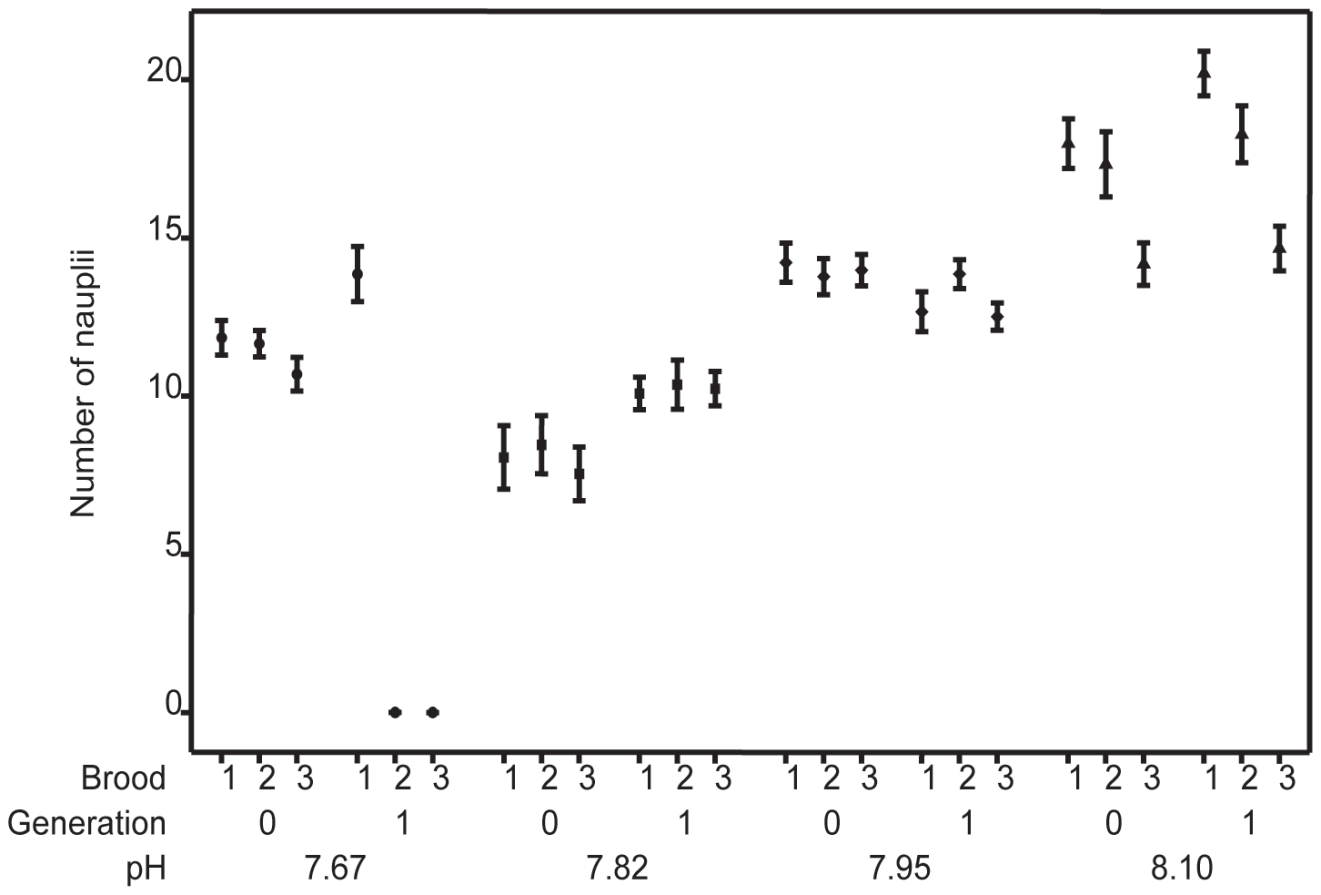
Naupliar production (mean ± SE, N = 32) for *Tisbe battagliai* cultured under specified ocean acidification scenarios with addition of supplementary copper. Data are grouped by brood and generation. Symbols; • = pH 7.67, ▪ = pH 7.82, ♦ = pH 7.95 and ▴ = pH 8.06.

**Figure 2:**
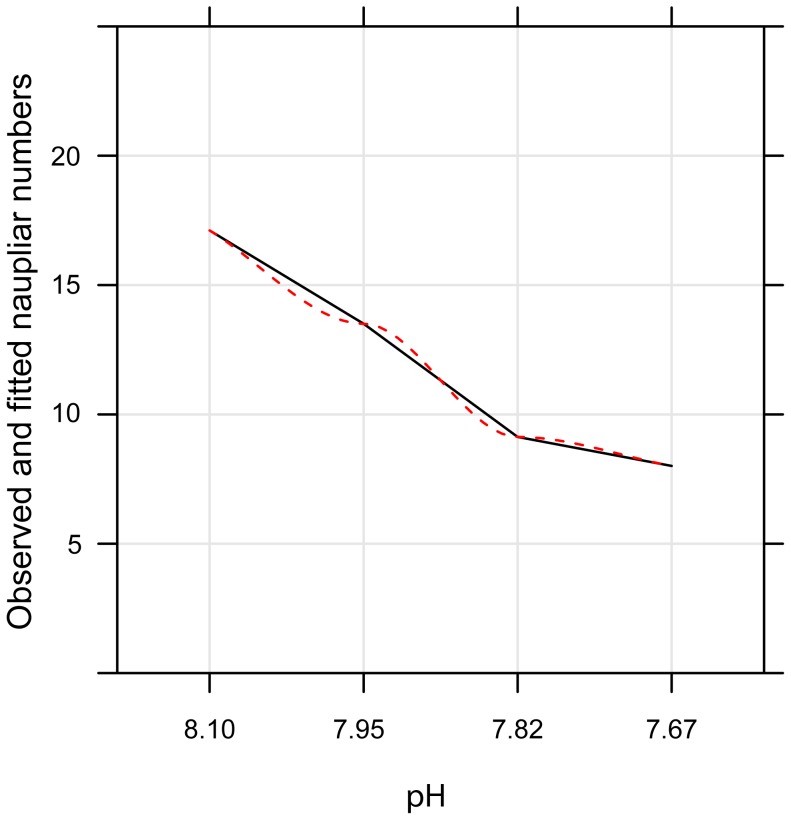
Model output for naupliar production data across pH 8.06, 7.95, 7.82 and 7.67 with addition of supplementary copper. Solid black lines represent actual data; dashed red lines represent observed model fit.

Naupliar production per brood and per generation under simulated ocean acidification conditions with elevated copper concentration is presented in Fig. 1. The data indicate a gradual decline in naupliar production with a reduction in pH from pH 8.06 (16.50±0.47) to pH 7.82 (8.03±0.66, *P*<0.001). A slight increase in the mean nauplair production for earlier generations at pH 7.67 (11.40±0.66 *P*<0.001) from pH 7.82, is reduced by mortalities in the later generations of pH 7.67 (8.50±0.93, *P*<0.001). Table 2 shows the GLS model output; the model predicts a gradual decline in naupliar production with decreasing pH below the control value (8.06), with mean naupliar production suppressed at all experimental pH conditions with the addition of copper. The largest decline occurred at pH 7.67, due to the inclusion of naupliar mortalities in the model. No significant difference was observed across generations when naupliar production was compared between the two generations for each pH with added copper. Comparison of interactions between pH and generation revealed significant interactions for pH 7.95 G1 (14.30±0.93, *t* = −2.36, *P = *0.018) and pH 7.67 G1 (8.50±0.93, *t* = −8.6, *P = *<0.001) (Table 2; Fig. 2).

**Table 2:** Random-effects variance components for the multi-level model.

Coefficient	Naupliar production number	Standard error	*t*-value	*P*-value
pH 8.06: Generation 0	16.50	0.47	35.47	<0.001
pH 7.95: Generation 0	13.99	0.66	−3.81	<0.001
pH 7.82: Generation 0	8.03	0.66	−12.88	<0.001
pH 7.67: Generation 0	11.40	0.66	−7.76	<0.001
pH 8.06: Generation 1	17.72	0.66	1.86	0.064
pH 7.95: Generation 1	14.30	0.93	−2.36	0.018
pH 7.82: Generation 1	17.48	0.93	1.05	0.292
pH 7.67: Generation 1	8.50	0.93	−8.6	<0.001

**Figure 3:**
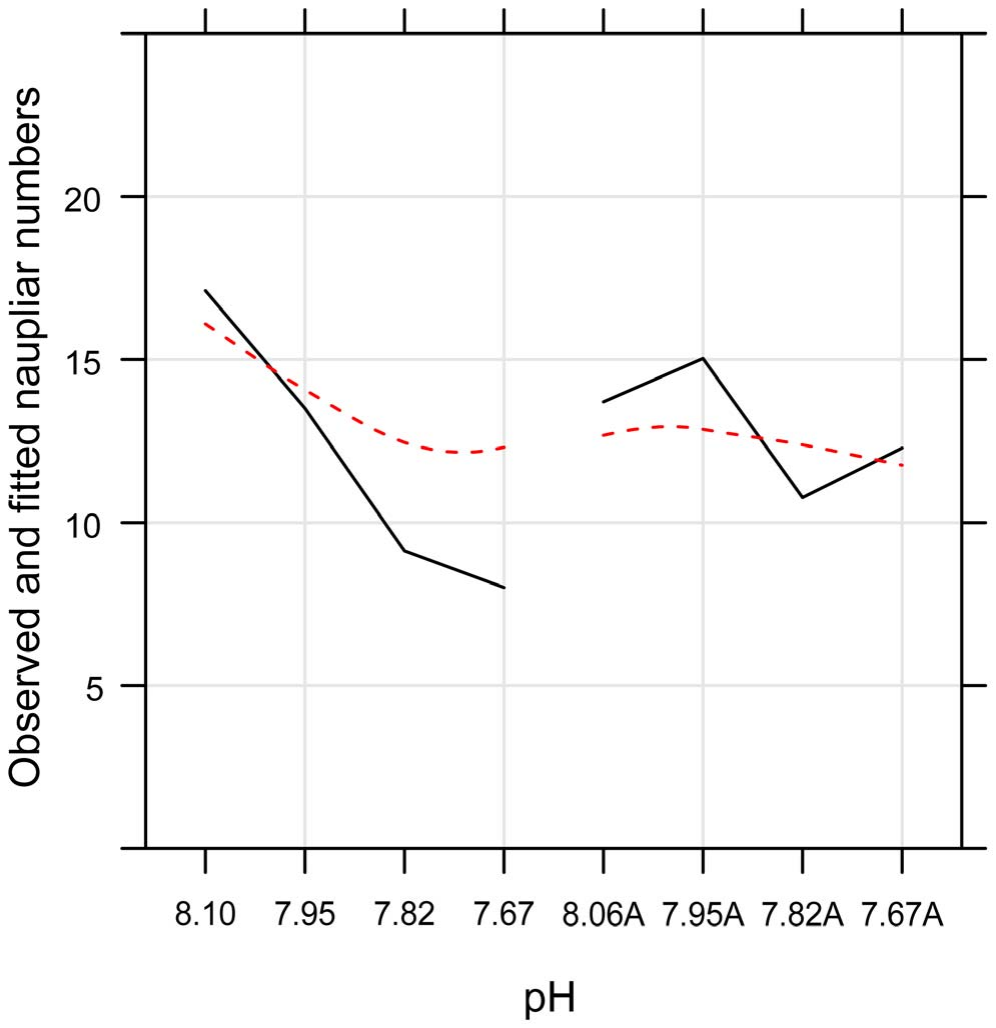
Model output for naupliar production across pH 8.06, 7.95, 7.82 and 7.67 with addition of supplementary copper and for conditions of simulated ocean acidification alone (pH 8.06A, 7.95A, 7.82A and 7.67A) across two generations, zero and one. Solid black lines represent actual data; dashed red lines represent observed model fit.

The second model examined all interactions, with and without the addition of copper (Table 3). Although our earlier data on the pH response alone covered three generations [16], only data for generations G0 and G1 were compared with data for pH and added copper from this work. The resulting model examined all naupliar production - pH interactions with and without copper addition. Naupliar production with decreasing pH and copper addition followed a significant further decline compared to decreasing pH alone (Fig. 3). Mean naupliar production for each pH with and without copper addition confirms that at pH 7.95 with copper addition (13.99±0.66, *t* = −3.71, *P = *<0.001) naupliar production was significantly lower than for the control pH 8.06, but also significantly lower than for pH 7.95 alone, which is not significantly different from the control pH alone (16.35±0.71, *t* = −0.22, *P* = 0.827) (Table 3). A further significant decline was observed from pH 7.82 to pH 7.67 as naupliar production for pH 7.82 and pH 7.67 plus copper was further reduced compared to the reduction in pH alone, suggesting an additive negative effect (Table 3, Fig. 3).

**Table 3:** Random-effects variance components for the multi-level model.

Coefficient	Naupliar production number	Standard error	*t*-value	*P*-value
pH 8.06: Generation 0	16.50	0.47	34.51	<0.001
pH 7.95: Generation 0	13.99	0.66	−3.71	<0.001
pH 7.82: Generation 0	8.03	0.66	−12.53	<0.001
pH 7.67: Generation 0	11.40	0.66	−7.55	<0.001
pH 8.06A: Generation 0	12.74	0.71	−5.32	<0.001
pH 7.95A: Generation 0	16.35	0.71	−0.22	0.827
pH 7.82A: Generation 0	10.47	0.71	−8.53	<0.001
pH 7.67A: Generation 0	12.14	0.71	−6.18	<0.001
pH 8.06: Generation 1	17.72	0.68	1.81	0.071
pH 7.95: Generation 1	14.30	0.96	−2.3	0.022
pH 7.82: Generation 1	17.48	0.96	1.03	0.305
pH 7.67: Generation 1	8.50	0.96	−8.37	<0.001
pH 8.06A: Generation 1	17.21	1.00	0.71	0.480
pH 7.95A: Generation 1	12.65	1.00	−3.85	<0.001
pH 7.82A: Generation 1	15.87	1.00	−0.63	0.530
pH 7.67A: Generation 1	15.58	1.00	−0.92	0.355

pH 8.06A, pH 7.95 A, pH 7.82A and pH 7.67A represent the same respective pHs alone without addition of copper.

### Naupliar Growth Analysis

Copepods grown with added copper did not differ significantly in length from the control pH 8.06 (349.48±8.96 µm) with declining pH, with the exception of pH 7.95 which was observed to significantly increase copepod length (446.13±9.6 µm, *t = *10.07, *P = *<0.001) (Table 4). Comparing growth data with and without added copper reveals a similar pattern, with a significant increase in copepod length at pH 7.95 [16]. All growth data for copepods grown at reduced pH with and without added copper were combined to produce an overall growth model (see model output Table 4). The model predicted a significant increase in copepod length at each pH following copper addition, revealing an additive positive impact from the addition of copper (Table 4).

**Table 4:** Generalised least square model output for naupliar growth data.

Coefficient	Length (µm)	Standard error	*t*-value	*P*-value
pH 8.06	349.48	8.96	39.01	<0.001
pH 8.06(no copper)	314.06	11.38	−3.11	0.002
pH 7.95	446.13	9.60	10.07	<0.001
pH 7.95(no copper)	341.20	11.01	−0.75	0.453
pH 7.82	366.13	10.12	1.65	0.101
pH 7.82(no copper)	302.04	10.92	−4.35	<0.001
pH 7.67	350.71	8.64	0.14	0.886
pH 7.67(no copper)	258.51	9.99	−9.11	<0.001
C3	353.63	9.65	0.43	0.667
C2	305.56	9.45	−4.65	<0.001
C1	286.36	9.85	−6.41	<0.001
N5	201.10	9.63	−15.41	<0.001
N4	132.71	10.35	−20.95	<0.001
N3	112.77	10.70	−22.11	<0.001

### Cuticle Elemental Composition

ESEM analyses identified the following elements in the copepod cuticle; carbon, oxygen, sodium, magnesium, aluminium, silicon, phosphorus, sulphur, potassium and calcium. Carbon and oxygen were the primary constituents comprising over half of the elemental content; the other elements were present only in trace quantities. Elements of interest (calcium, phosphorus and sulphur), observed in copepod cuticle were compared between exposure to reduced pH with and without copper [16]. The carbon and oxygen mass percentages were not significantly different when comparing pH 8.06 to pH 7.95 and pH 7.67. However, at pH 7.82 cuticle carbon and oxygen were, respectively, significantly lower (12.36±8.31, ANOVA *P* = <0.001) and significantly higher (70.91±6.10, ANOVA *P* = <0.001) with respect to the control pH (mean carbon, 41.14±9.23; mean oxygen, 55.17±9.81, errors represent ± one standard error). Differences were observed between the elements of interest in the cuticle, particularly with respect to sulphur, phosphorus and calcium (Table 5). These elements were compared between the experimental pH treatments with and without copper addition (Table 5). Significantly higher phosphorus, sulphur and calcium were observed at pH 7.82 (phosphorus 0.61±0.22; sulphur 1.16±0.55; calcium 0.94±0.20; all ANOVA P = <0.001) and significantly higher phosphorus and calcium were also observed at pH 7.95 (phosphorus 0.48±0.11; calcium 0.47±0.04; both ANOVA *P* = <0.001) relative to the control (phosphorus 0.28±0.06; sulphur 0.61±0.13; calcium 0.21±0.04; all ANOVA *P* = <0.001). Comparison of the same elements at each pH with and without copper addition showed significantly less phosphorus at pH 7.82 from pH 7.82 alone (0.87±0.23, ANOVA *P* = <0.001) and a decrease, although not significant, at pH 7.67 and 7.95 with copper addition from pH 7.67 alone (0.25±0.06) and pH 7.95 (0.54±0.22) alone. All pH treatments with added copper showed significantly reduced phosphorus relative to the control pH 8.06 with copper (0.57±0.11, ANOVA P = <0.001). No significant changes or patterns were observed for sulphur, however there were significant decreases in calcium at pH 8.06 from pH 8.06 alone (0.66±0.52, ANOVA P = <0.001), 7.95 from pH 7.95 alone (1.34±0.62, ANOVA P = <0.001) and 7.67 from pH 7.67 alone (0.51±0.20, ANOVA P = <0.001) with added copper.

**Table 5:** Mean copepod cuticle elemental concentrations given as means normalised to carbon by weight percentage ± one standard error.

Element	pH	No added copper	Added copper
Carbon	8.06	41.70±8.99	41.14±9.23
	7.95	37.15±4.52	36.09±2.24
	7.82	43.28±4.11	12.36±8.31
	7.67	52.55±1.38	49.52±1.37
Oxygen	8.06	52.51±8.32	55.17±9.81
	7.95	50.71±0.65	55.03±1.68
	7.82	48.23±2.95	70.91±6.10
	7.67	43.40±0.59	47.43±1.30
Phosphorus	8.06	0.59±0.12	0.28±0.06
	7.95	0.54±0.25	0.48±0.11
	7.82	0.87±0.10	0.61±0.22
	7.67	0.25±0.04	0.22±0.10
Sulphur	8.06	0.69±0.15	0.61±0.13
	7.95	0.40±0.06	0.51±0.06
	7.82	0.64±0.34	1.16±0.55
	7.67	0.38±0.15	0.44±0.07
Calcium	8.06	0.81±0.19	0.21±0.04
	7.95	1.85±0.88	0.47±0.04
	7.82	0.70±0.15	0.94±0.20
	7.67	0.50±0.18	0.21±0.05

### Copepod Copper Uptake with Decreasing pH and Addition of Copper

Elemental examination of the copepod cuticle revealed no copper presence. To confirm copper uptake by the copepods, complete copepod digests were performed. Gravid females from the first generation for each pH were used. Unfortunately, due to mortalities during the pH 7.67 experiments insufficient females were available for digestions. Comparisons were made between copepods grown at pH 8.06 with copper, to those copepods grown at pH 8.06 without copper and pH 7.95 and pH 7.82 with copper. An increase in copper concentration was observed for pH 7.82 and pH 7.95 from the control (undetectable) to 10 µg L^−1^ of copper per 10 gravid females.

## Discussion

Ocean acidification does not pose an environmental threat in isolation. The factors behind ocean acidification, principally an increase in the atmospheric carbon inventory, will drive additional environmental change such as elevated sea surface temperature and ocean hypoxic events. These factors, when considered alongside environmental co-stressors such as chemical contamination, must be considered collectively if we are to achieve a true understanding, and therefore attain greater predictive capacity, of the responses of the marine environment to climate change. Given the logistical and analytical difficulties associated with conducting long term, multi-stressor and multigenerational studies, we have instead chosen to combine ocean acidification and background copper exposure as cofactors over a number of generations. Our results imply that increasing ocean acidification has the potential to turn environmentally relevant concentrations of copper to acute or potentially lethal concentrations impacting on benthic copepod reproduction and development.

### Naupliar Production Response to Combined Ocean Acidification and Environmental Copper

The majority of previous studies examining the effects of ocean acidification as a co-stressor have so far focused on temperature [27]–[30], [32]–[35], [37]. These short-term studies have thus far proved inconclusive; further, it is becoming evident that often the impact of the co-stressor (particularly temperature) greatly outweighs the negative impacts attributable to ocean acidification, for example see Byrne 2009 [29]; although the picture remains not entirely clear. Very few ocean acidification studies have considered trace metal exposure as a factor; those that have tended to use either severely reduced pH conditions in the context of CO_2_ leakage from sub-sea storage (pH 6.07–6.36) [36], failed to consider the vital importance of exposure duration, particularly across multiple generations [57], or used inappropriately high concentrations of metal contaminants [58]. Nevertheless, it is essential to consider how ocean acidification interacts with marine contaminants and xenobiotics [59].

Copper is known to be toxic to reproductive processes in copepods [39]–[40], [60]–[61], and enhanced toxicity has been observed when combined with ocean acidification [36]. Pascal et al. 2010 [36] noted an antagonistic effect of ocean acidification and copper on mortality of the harpacticoid copepod *Amphiascoides atopus*, demonstrating the importance of an increase in free copper ions. This is in broad agreement with the main findings of this study. However, it must be stressed that we have exposed three generations using substantially more conservative pH levels combined with background copper concentrations. Here, combined ocean acidification and environmental copper amplified the impact on naupliar production over multiple generations indicating future copepod population decline.

### Growth Response to Combined Ocean Acidification and Environmental Copper

Previously, Fitzer et al. 2012a&b [16], [62] observed a marked reduction in somatic growth for *T. battagliai* cultured under ocean acidification scenarios without a co-stressor. This was coupled with an overall decline in reproductive output, despite the animals appearing to reallocate resources to maintain high levels of fecundity. However, with copper as a co-stressor, there was a significant increase in growth at each pH. Naupliar production was observed to decline overall as in the previous study, yet without any apparent attempt to reallocate additional resource to reproduction. This contrasting growth response indicates that, in this particular situation, *Tisbe* are preferentially reallocating energy to somatic growth at the expense of fecundity. Stump et al. 2011 [63] observed developmental delay in sea urchin larvae alongside increased metabolic rates, suggesting that changes in energy budgets may result from ocean acidification stress leading to increased scope for growth; however, no such pattern was observed by Matson et al. 2012 [64]. An alternative explanation may be that under the culture conditions, the animals were copper deficient and by increasing copper bioavailability through pH manipulation we have thereby released *Tisbe* from a growth limiting condition. Copper deficiency in copepods is not unknown, for instance Lundström et al. 2010 [65] suggested that activated carbon filtration may induce essential metal limitation by the removal of metal ions from culture waters. Dave 1984 [66] exposed *Daphnia magna* to a range of copper concentrations, observing induced phases of stimulatory growth in juveniles, although these increases had been negated upon reaching adulthood. There were also phases in which reproductive output was substantially enhanced. Dave 1984 [66] notes that the response to the provision of an essential yet limiting element is often observed phenotypically as growth stimulation; however, growth, or the stimulation of other biological processes may be equally attributable to a hormesis-type response [67]. Copper has been linked to similar hormesis responses in hydroids as well as in earlier studies on *D. magna* [68]–[70]. Fitzer et al. 2012 [16] attributed the growth and reproductive patterns expressed by *T. battagliai* under ocean acidification conditions to a hormetic response; however, in the current study it was not possible to entirely distinguish between the role of copper as either an inducer of hormesis and its role as an essential nutrient. It is however, pertinent to consider the concept of the window of essentiality, or alternatively the optimal concentration range for essential elements (OCEE) as described by Van Assche et al. 1997 [71] and Hopkin 1989 [72]. The model describes a bell-shaped curve with elemental deficiency at the extreme left hand side, elemental toxicity to the extreme right and the OCEE between the two. One may consider the situation whereby copepods cultured without supplemental copper, or indeed with copper at ambient pH, are in the deficient section and as declining pH increases copper bioavailability the model stasis moves to the right into the OCEE region, as evidenced by enhanced growth. Further, as pH continues to drop the stasis continues to shift to the right and begins to encroach into the toxic range, as evidenced by the declining reproductive output. Such a scenario is described for *D. magna* by Bossuyt & Janssen 2004 [73]. Whereas this concept of stimulated growth may potentially be beneficial at the individual level [74], the overall consensus remains that reproduction, and therefore population dynamics, will suffer under acidified conditions, with or without the burden of copper exposure. We therefore draw the conclusion, from a population perspective, that under ocean acidification scenarios the essential element status of copper is driven into the toxic range of the window of essentiality.

Copper uptake may have been direct, but more likely would have been via the food source. Ocean acidification conditions have been demonstrated to constrain microalgae fatty acid profiles, which may impact energy transfer along trophic levels [75]. It could be argued that such a situation may have occurred in the Fitzer et al. 2012 [16] study wherein the nutritional quality of the microalgae diet would have been *de facto* downgraded; hence the observed reduction in somatic growth. Conversely, supplementing copper may have upgraded the nutritional status of the diet by increasing protein and lipid titres [76]. Similarly, the possibility remains that the microalgae may have complexed the free copper ions and therefore reduced their toxicity [77]. Notwithstanding these points, our data are for copepods fed *ad libitum* on a mixed microalgae diet and although clearance rates were not recorded, justification for growth due to algal quality remains speculative.

### Cuticle Composition Response to Environmental Copper and Ocean Acidification

Cuticle elemental composition using ESEM-EDX was employed as a means to examine copper uptake, yet interestingly, when comparing cuticle composition between copper supplemented and non-supplemented experiments, copper was undetected. There were, however, significant decreases for sulphur, phosphorus and calcium at all pH values. Copper is taken into cells through binding with transport ligands (proteins in the proteinaceous matrix) to allow a route across the hydrophobic cell membrane [78]. These internal ligands are likely to have *S*- and *N*-binding sites, ready to accept trace metals, containing protein functional groups [78]. Although copper-sulphur granules are involved in the metabolism of haemocyanin and are related to the moult cycle in crustaceans [79], this is not observed in copepods; they are more likely to have a role in detoxification [79]. The decrease in sulphur concentrations within the cuticle implies that more copper is being taken up through the copepod cuticle under acidified conditions. Once copper has entered the cell, concentrations exceeding the organism’s requirement need to be detoxified; it is more dangerous for the organism to be exposed to lower concentrations of metabolically available metals than a higher burden of accumulated but detoxified metals [78]. Metallothioneins, present and functional in copepods [80] play an important role in redistributing copper; subsequently they are involved in lysosomal breakdown, leaving free insoluble metal-rich compounds [78]. These compounds bind to calcium-containing granules based on phosphate which plays a further detoxifying role [78]. The decrease in the cuticle burden of both phosphorus and calcium in all pH plus copper treatments compared with pH alone suggests that phosphorus and calcium removal from the cuticle may be functioning as a mechanism of copper detoxification in this system. In polluted systems it has been shown that phosphorus and sulphur, along with trace metals, can accumulate in the residual lysosomes of gastropod digestive cells [79]. Copepods have also been shown to have a high tolerance for copper as one of the essential trace metals, and at low levels of copper, metallothionein-like proteins are induced to cope with increased trace metal stress [26]. In combination with the reduced naupliar production it would seem that increasing ocean acidification will likely cause environmentally relevant concentrations of copper uptake to become toxic to benthic copepods. Confirmation of copper up take was achieved through complete digestion of copepods further supporting this hypothesis.

### Possible Implications for Benthic Copepods

Our results for *T. battagliai* suggest that increasing ocean acidification in concert with environmentally realistic copper concentrations will prove detrimental to copepod reproduction and development. Direct impacts may involve a possible energy trade off with naupliar production and somatic growth as a direct result of environmental stress. Nevertheless, this remains a topic that is subject to considerable debate and interpretation. Despite the multigenerational aspect of this study and that of its forerunner [16], it is as yet unclear whether *Tisbe*, as a representative copepod, is capable of acclimation to an acidified and potentially more toxic ocean. It is however patently clear that *Tisbe* has the capacity for significant phenotypic plasticity as evidenced by its plastic approach to growth. What is unclear is at which point this plasticity begins to progress to acclimation and adaptation [81].

Further study into scope for growth with combined stressors is vital to explore the underlying mechanisms in greater detail. Multigenerational modelling predicted a population decline in *T. battagliai* with future projections of ocean acidification combined with environmental copper concentrations beyond that of ocean acidification impact alone. Thus, in the future ocean copepods may be both larger and smaller in number, presenting problems to predators such as juvenile fish. The adoption of a universal view on ocean acidification impacts seems both unlikely and unjustified. The “one size fits all” approach to ocean acidification research does not take into account local systems or regional variability. Individual laboratory experiments examining ocean acidification impact on marine organisms have been shown to have very different impacts compared to impacts combined with increased temperature. Further investigation of the combined impact of benthic environmental stressors and ocean acidification on benthic marine organisms is required to further predict the natural ecosystem impact of decreasing ocean pH.

## Supporting Information

File S1 (XLS)

File S2 (XLS)

File S3 (XLS)
